# Imaging Features of Symptomatic Hypertrophic Tuberculum Peroneum

**DOI:** 10.5334/jbr-btr.1376

**Published:** 2017-12-16

**Authors:** Julie Desimpel, Magdalena Posadzy, Filip Vanhoenacker

**Affiliations:** 1 AZ Sint-Maarten Duffel/Mechelen and University (hospital) Antwerp, Edegem, BE; 2 Dega Orthopaedic and Rehabilitation University Hospital, PL; 3 Karol Marcinkowski University of Medical Sciences, Poznan, PL; 4 AZ Sint-Maarten and University (Hospital) Antwerp/Ghent, BE

**Keywords:** Hypertrophic tuberculum peroneum, Ultrasound, MRI, (CB)CT

## Abstract

**Objective:**

The aim of this article is to review the clinical and imaging features of symptomatic hypertrophic (TP) in a cohort of symptomatic patients.

**Materials and Methods:**

Twenty-three patients with chronic lateral ankle pain were retrospectively included in our study group. Patients underwent ultrasound (US), (cone beam) computed tomography (CB)CT or magnetic resonance (MR) examination or a combination of these examinations with a standardized protocol. Patients with an underlying fracture were excluded. The following parameters were recorded: clinical history, size of the TP on different imaging modalities, presence and grade of peroneus brevis/longus tenosynovitis and the presence of bone marrow edema at the os calcaneus on magnetic resonance imaging (MRI).

**Results:**

The mean width of the hypertrophic TP was 5.6 mm. Combined tenosynovitis of the peroneus longus (PL) and brevis tendon (PB) was most common, followed by isolated PL and finally PB tenosynovitis. Grade 1 tenosynovitis was most common. BME was present in 53% of the cases.

**Conclusion:**

The width of the TP is may be evaluated on the (oblique) coronal US, (CB)CT or non-fat suppressed MR images. Both US and MRI may detect and grade involvement of the peroneal tendons. By the use of fluid sensitive sequences, MRI may be of additional value to detect bone marrow edema as result of repetitive friction.

## Introduction

Ankle pain may result from a variety of diseases. An underestimated cause of chronic lateral ankle pain is the presence of a hypertrophic tuberculum peroneum (TP) [1]. As the differential diagnosis with more frequent causes of ankle pain solely based on clinical presentation is often impossible, imaging is very useful for evaluation of the size of a TP and its effect on the surrounding structures.

**Figure 1:**
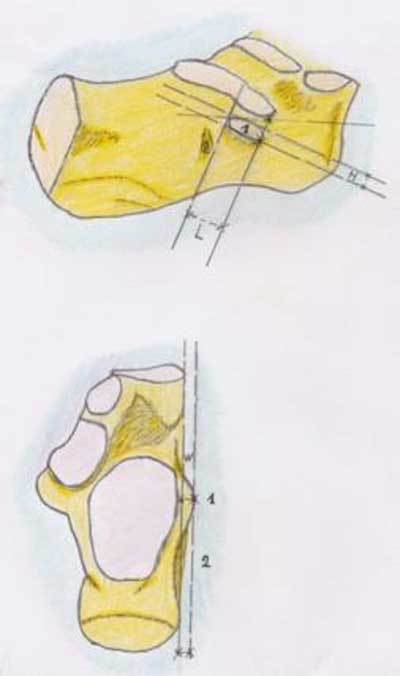
Schematic drawing of the tuberculum peroneum and its size. Lateral and superior view of the tuberculum peroneum (1) which is located anteriorly to the eminentia retrotrochlearis (2) on the calcaneus. The length (L) and height (H) are best visualized on oblique sagittal or coronal cross-sectional images. The width is the distance between the tangent line between the anterior and posterior cortex of the calcaneus and the most laterally located cortex of the TP. This parameter is easily measured on either axial or coronal cross-sectional imaging methods.

The TP is located on the lateral surface of the calcaneus, anteriorly to the eminentia retrotrochlearis (Figure 1). Anatomically, the TP has an oblique course from postero-superior to antero-inferior. The peroneus longus and brevis tendon run respectively under and above the TP, separating their tendon sheaths. It functions as fulcrum directing the peroneus longus tendon towards the cuboid. Furthermore, the inferior peroneal retinaculum inserts on the TP [2].

**Figure 2:**
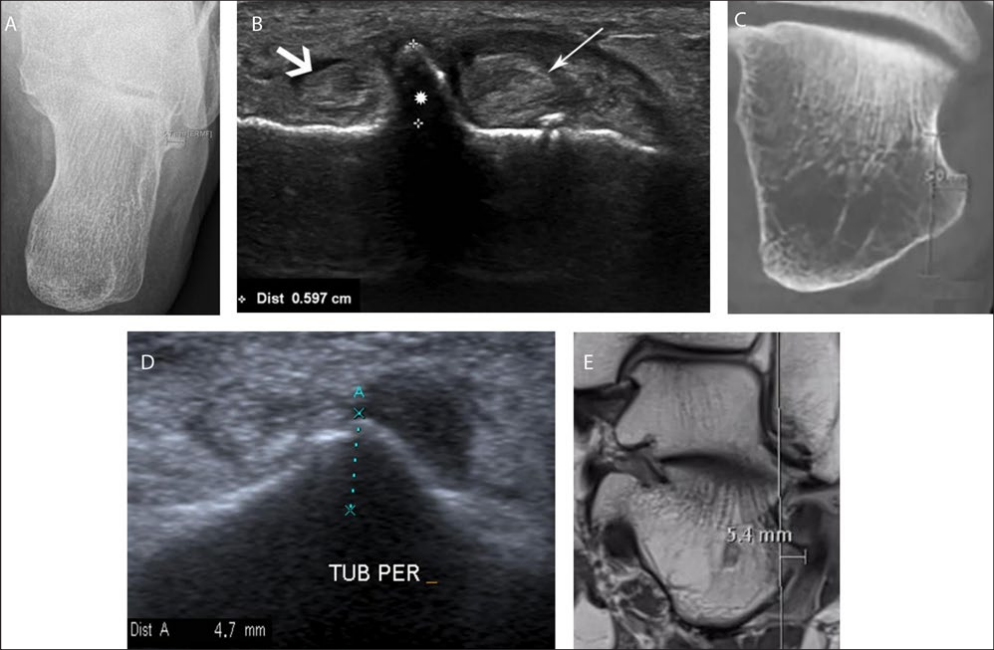
Examples of measurement of the width of the TP on different imaging methods. Axial CR **(A)** shows a hypertrophic TP with a maximum width of 5.7 mm measured between the base and the top of the tuberculum. Oblique coronal US **(B)** confirmed the presence of a hypertrophic TP (white asterisks) with a measured width located between the course of the peroneus longus (thin white arrow) and brevis (thick white arrow). Coronal CBCT **(C)** reveals a hypertrophic tuberculum peroneum in another patient with a width of 5 mm. This measurement correlates well with the width measured on the oblique coronal ultrasound in the same patient (**D**). Coronal PD MR image demonstrating the width measurement of the hypertrophic tuberculum peroneum **(E)**.

Its size is defined by width, height and length. Because conventional radiography (CR) is usually not accurate for precise evaluation of the size of the TP, measurements are far better performed on ultrasound (US), (CB)CT or MRI [3] The width of the TP is the easiest parameter to evaluate (Figure 2) on oblique coronal US images and on axial or coronal CT or MR images (Figure 2). Hyer, et al. [3] examined human skeletons focusing on the characteristics of the tuberculum peroneum. Their measurements revealed an average width of 3.13 mm.

Hypertrophy of the TP will cause friction and mechanical irritation which may lead to peroneal tenosynovitis causing lateral ankle pain. Anatomically, the peroneus longus tendon is most at risk for tenosynovitis because of its long excursion and changing direction. The peroneus brevis tendon is less prone to be involved [4,5].

Despite multiple previous case reports and small studies, a uniform definition of a hypertrophic tuberculum peroneum is still debated. Based on those small reports a width exceeding 5 mm is considered as being hypertrophic predisposing to tenosynovitis [6].

The aim of this article is to review the clinical and imaging features of symptomatic hypertrophic (TP) in a cohort of symptomatic patients.

## Material and Methods

### Patients

Our study is a monocentric, retrospective cohort study of 23 patients. Inclusion criteria included referral for imaging (either US, (CB) CT or MRI; Table 1) for chronic lateral ankle pain below the lateral malleolus. Patients with underlying fracture were excluded.

**Table 1:** Overview of imaging modalities used in our series.

Type of examination	Number of patients

X-ray	10
US	12
CBCT	2
MRI	17

### Imaging protocols

**Figure 3:**
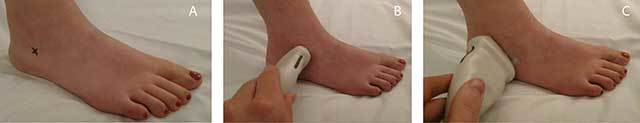
Clinical pictures of the position of the US transducer. The X on the lateral side of the foot **(A)** represents the anatomical location of the TP, **(B)** and **(C)** show the position of the ultrasound transducer in oblique coronal and oblique axial position, respectively, for accurate measurement of the width of the TP.

Conventional radiography was performed on the Luminos dRF MAX (Siemens, Erlangen, Germany). AP, lateral and Mortise view are the three standard images performed of the ankle. Additional axial image to visualize the hypertrophic tuberculum was performed. US images using EPIQ5G (Philips Health Systems, Bothell, WA 98021, USA) (Figure 3). Cone Beam CT (CBCT) imaging was performed on a Newtom 5G-system (QR, Verona, Italy), with a field of view of 8 × 8 cm, centered on the painful ankle region. MR examinations were performed on a 1.5T system (Siemens, Magnetom Aera, Erlangen, Germany). Our routine protocol consisted of sagittal, axial and coronal fat suppressed (FS) T2-Weighted Images (WI), coronal PD and axial T1-WI with a slice thickness of 3 mm. Only seven patients were examined by one imaging modality (MRI). The other patients had at least a combination of two imaging techniques of which the combination of MR and US was most frequent (6 patients or 26%).

### Analysis of clinical history and imaging parameters

The following parameters were retrospectively reviewed by two observers in consensus: history of previous ankle sprain or trauma, width of the TP on all available imaging modalities, the presence and grade (I–III) [7] of tenosynovitis of peroneus brevis (PB) and/or longus tendon (PL) (Table 2), bone marrow edema (BME) at the os calcaneus.

**Table 2:** Tenosynovitis grading according to reference [7].

Grade 1	Focal thickening of the tendon + effusion
Grade 2	Partial thickness tear
Grade 3	Full thickness tear

## Results

Twenty-three patients were included in our study, 10 men and 13 women. Eleven patients presented with right-sided chronic ankle pain while the left ankle was affected in 12 cases.

Nine patients had a history of previous ankle distortion, ranging between several months to years previous to presentation. In four patients the request form for imaging mentioned overuse. One patient had planovalgus foot deformity.

**Figure 4:**
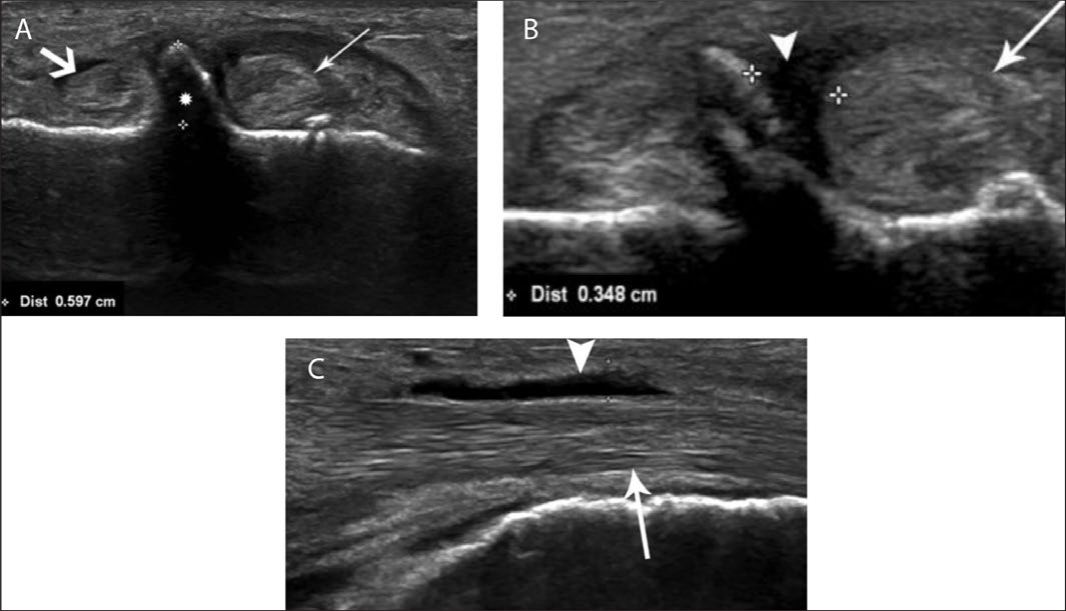
Hypertrophic TP and grade 1 tenosynovitis of the peroneus longus in a 67-year-old male. Oblique coronal (a and b) and longitudinal (c) US. Oblique coronal US **(A)** Reveals a hypertrophic TP (white asterisks) with a measured width of 6 mm located between the peroneus longus (thin white arrow) and peroneus brevis (thick white arrow). Note also surrounding effusion in the tendon sheath of the PL (white arrowhead) **(B)**. On longitudinal ultrasound imaging **(C)** Thickening of the peroneus longus (thin white arrow) is seen with surrounding fluid (white arrowhead).

**Figure 5:**
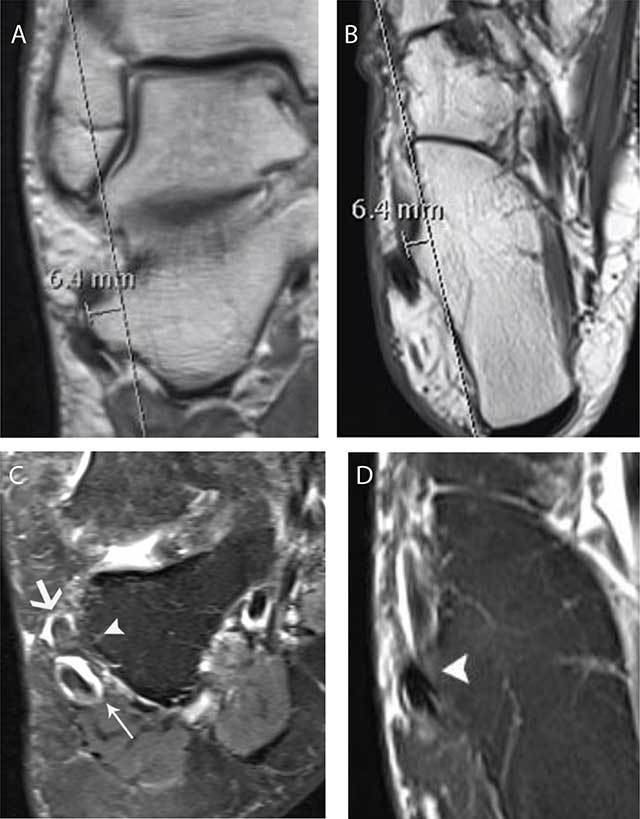
Hypertrophic TP and grade 1 tenosynovitis of the peroneus longus and brevis in a 64-year-old woman. Coronal PD **(A)**, axial T1-WI **(B)**, coronal FS T2-WI **(C)** and axial FS T2-WI **(D)** MR images. The hypertrophic tuberculum peroneum is measured on the coronal (a) and axial (b) PD images and has a width of 6.4 mm. The size is best measured on non-FS images as the cortical delineation is more precise than on the FS T2-WI. There is a focal thickening of the peroneus longus tendon (thin white arrow) with increased signal and effusion on the coronal (c) FS T2-WI MR images. Note also increased fluid within the tendon sheath of the peroneus brevis (thick white arrow) in keeping with mechanical friction. There is subtle bone marrow edema at the TP (white arrowhead). The axial (d) FS T2-WI MR images reveal also subtle bone marrow edema (white arrowhead).

The mean width of the TP in these symptomatic patients was 5.6 mm (range 3.6–8.6 mm). Isolated tenosynovitis of the peroneus longus (Figure 4) was seen in nine cases (grade 1 n = 6 and grade 2 n = 3). Isolated tenosynovitis of the peroneus brevis was present in only 4 patients (grade 1 n = 2 and grade 2 n = 2). Concomitant involvement of both peroneal tendons (Figure 5) was seen in 10 patients (Table 3). BME is was solely visible on the FS T2-WI images and was present in 53% (9 out of 17 patients).

**Table 3:** Overview of the combination of peroneal tendon involvement.

Type of concomitant tenosynovitis	Number of patients

Grade 1 tenosynovitis brevis and grade 1 tenosynovitis longus	3
Grade 1 tenosynovitis brevis and grade 2 tenosynovitis longus	2
Grade 2 tenosynovitis brevis and grade 1 tenosynovitis longus	2
Grade 2 tenosynovitis brevis and grade 2 tenosynovitis longus	3

## Discussion

Hypertrophy of the tuberculum peroneum is an underdiagnosed cause of chronic lateral ankle pain. Detailed anatomy of the calcaneus and the TP was previously described by Laidlaw [8] and Edwards [9] and Hyer, et al. [3]. Although the literature does not provide a clear definition about the size of a hypertrophic TP in large studies, a width ≥5 mm is considered as being hypertrophic [6]. This correlates with the mean width measured in our symptomatic patient population.

The pathogenesis of a hypertrophic TP is variable and controversial. Some authors consider a hypertrophic tuberculum peroneum as a pre-existing congenital abnormality [10], whereas others believe it is a secondary finding due to repetitive and excessive traction on the peroneal tubercle [11]. The latter hypothesis is supported by the review of the clinical history in our study population. Nine patients had a previous ankle distortion, which may have led to chronic microinstability due to ligamentous damage. In another four patients, a history chronic overuse was present.

Symptomatic patients complain of pain at the lateral side of the ankle. In acute posttraumatic setting local swelling may be present. There may be concomitant painful palpation and inversion as well.

Friction with the peroneal tendons cause local attrition and tendon degeneration. The grade of tenosynovitis varies from focal thickening of the tendon and local effusion to a partial thickness tear or even complete rupture [7]. Because of its anatomical predisposition, the peroneus longus tendon was believed to be the most frequently affected tendon in case of hypertrophy of TP [4,5]. The peroneus longus tendon was also involved in 83% of our population [4,5].

Only a few cases of tenosynovitis of the peroneus brevis have been described in the literature [12]. However, in our series tenosynovitis of the peroneus brevis was seen in 14 patients or 61% (either isolated and concomitant).

Although there is currently no standardization on the measurement of TP on different imaging techniques, we recommend ultrasound as the initial imaging modality in case of suspicion of a symptomatic TP and MRI for more precise locoregional evaluation, demonstration of associated BME at the calcaneum and especially for differential diagnosis (Figure 5) for other causes of lateral ankle pain.

Due to its complex anatomy and superposition of osseous structures, plain radiography is inaccurate for evaluation of a TP.

(CB)CT allows more precise measurement compared to plain radiography, but there is a concern about radiation exposure. Moreover, if CBCT is used, there is insufficient visualisation of surrounding soft tissues.

Ultrasound allows precise correlation with the location of the pain of the patient. Evaluation of the width of the TP is readily done on oblique coronal images. The dynamic capability of this modality is a major advantage to evaluate real time peroneal tendon impingement or clicking. Ultrasound allows also accurate staging of the grade of tenosynovitis with a very high spatial resolution.

Whilst MRI lacks the dynamic aspect of ultrasound, it allows visualization of BME at the calcaneum caused by repetitive friction of the peroneal tendons against the adjacent bone.

The best sequences to document BME are fluid-sensitive sequences, such as STIR or fat suppressed T2-WI. In our series, BME was seen in 53% of symptomatic patients. For evaluation of the width of the TP, non-fatsuppressed coronal or axial PD or T1-WI are preferred, because they allow better visualisation of the cortical margins of the TP.

A significant limitation of our study is the retrospective design, which may result in selection bias, as a hypertrophic tuberculum peroneum may have been present in other asymptomatic individuals not seeking medical assistance. Furthermore, due to its retrospective design, every single patient was not examined by every imaging modality, which renders strict correlation of the imaging modalities impossible. Note also that the routine MR protocol in our institution includes strict coronal, axial and sagittal 3 mm slices, which are not corrected for the oblique anatomical orientation of the TP, which may be the origin of subtle mismatch of measurements on ultrasound compared to MRI. The second limitation is the limited number of patients. However, in comparison to the previously published literature, this is one of the largest series in symptomatic patients. The fact that the measurements were performed by 2 observers in consensus and not independently is another limitation of the study.

Therefore, further studies are needed to define the size of the TP in a large cohort of asymptomatic individuals and to correlate those measurements on different imaging modalities and slice orientations. Calculation of inter- and intra-observer variability will be useful to find out whether imaging techniques are reliable in assessment of the size of TP and for further refinement of the diagnostic algorithm of chronic lateral foot pain.

## Conclusion

Hypertrophy of TP should be considered as a potential origin of chronic lateral ankle pain.

Ultrasound may be used as screening modality in the diagnosis by measurement of the width on coronal oblique images and evaluation of the peroneal tendons. Associated BME is best detected on the fatsuppressed T2-WI MR or STIR images, in combination with T1-WI or PD images for evaluation of the size of the TP.
